# Development and multi-site validation of a new condition-specific quality of life measure for eating disorders

**DOI:** 10.1186/1477-7525-5-23

**Published:** 2007-04-30

**Authors:** Carol E Adair, Gisele C Marcoux, Brian S Cram, Carol J Ewashen, Janet Chafe, Stephanie E Cassin, Jorge Pinzon, Joanne L Gusella, Josie Geller, Yvette Scattolon, Patricia Fergusson, Lisa Styles, Krista E Brown

**Affiliations:** 1 Departments of Community Health Sciences and Psychiatry, Faculty of Medicine, University of Calgary, Room 124 Health Sciences Centre, 3330 Hospital Dr. NW, Calgary, AB T2N 4N1, Canada; 2 Calgary Health Region, 10101 Southport Road SW, Calgary, AB, T2W 3N2, Canada; 3 Faculty of Nursing, University of Calgary, 2500 University Dr. NW, Calgary, AB T2N 1N4, Canada; 4 Children's and Women's Health Centre of British Columbia, 4500 Oak Street, Vancouver, BC, V6H 3N1, Canada; 5 Faculty of Medicine, University of British Columbia, Vancouver, BC, Canada; 6 Dalhousie University, Halifax, Nova Scotia, B3H 3J5, Canada; 7 Providence Health Care, 1081 Burrard Street, Vancouver, BC, V6Z 1Y6, Canada; 8 Capital Health, QEII Health Sciences Centre,1278 Tower Road, Halifax, Nova Scotia, B3H 2Y9, Canada; 9 University of Manitoba, Winnipeg, Manitoba, R3T 2N2, Canada

## Abstract

**Background:**

In eating disorders (EDs) treatment, outcome measurement has traditionally focused on symptom reduction rather than functioning or quality of life (QoL). Generic QoL measures lack sensitivity for some diagnoses and many not be responsive in eating disorder patients. This article describes the development and validation of a condition-specific QoL measure for adolescents and adults with eating disorders – the Eating Disorders Quality of Life Scale (EDQLS).

**Methods:**

Multi-source and multi-stage methods were used to develop the EDQLS, with participation of patients with EDs, their family members and ED treatment providers. Sources for domain and item development included 39 articles, 12 patient and 10 treatment provider interviews, and 31 first person narratives from the internet. Four stages of validation and pre-testing involving 17 patients, 10 family members and 18 providers reduced 233 items to 40 items in 12 domains. These items were pilot tested in 41 ED patients.

**Results:**

The final instrument was then validated in a 12 site sample of 171 individuals aged 14–60 with EDs. All items showed good dispersion. The total raw mean score was 110 out of 200 (SD 27.6) with higher scores indicating better QoL. Internal consistency was excellent (Cronbach's alpha = .96) and subscale internal consistency ranged from alpha .36 to .79 providing evidence for a strong overall construct and some multi-dimensionality. Validity was supported by significant differences in mean EDQLS according to severity levels on the EDI-2 (F = 95.3, p <.001) and the BSI (F = 86.9, p <.001). EDQLS scores were positively associated with time in treatment (F = 4.65, p = .01) suggesting responsiveness. A strong positive association was also found between EDQLS scores and stage of change (F = 15.1 p <.001). Pearson's correlations between the EDQLS and criterion instrument scores were .71 for the SF-12 mental subscale, .61 for the QoLI and .78 for the 16D, supporting construct validity. Exploratory principal components and item response theory analyses identified only a few poor fitting items.

**Conclusion:**

The EDQLS has promising psychometric characteristics and may be useful for evaluating ED treatment effectiveness.

## Background

In an increasingly appearance obsessed society, eating disorders (EDs) including anorexia nervosa (AN), bulimia nervosa (BN), and eating disorders not otherwise specified (EDNOS including binge eating disorders) represent a serious health threat to children, youth, and adults of both sexes [1]. Recent population-based data provide stronger evidence of increased prevalence of EDs in recent birth cohorts, and confirm that only a minority of cases have received treatment [1]. Increased prevalence of unhealthy dieting behaviors that elevate risk for the development of EDs is also disturbing. Recently published studies document disordered eating attitudes and behaviors in 27–29% of girls aged 10–18 years and increases in concern with weight over time among boys and girls aged 9–14 [2-4]. These trends imply that EDs will continue to be a significant health concern for the foreseeable future.

If not treated early and effectively, EDs can become chronic, and place enormous burden on the patient and his/her family [5]. Demand for treatment services is growing along with an urgency to ground new treatments in evidence [6,7]. Treatment outcome measurement in EDs has traditionally focused on changing behavior and symptoms (e.g. reducing purging or achieving healthy body weight) rather than on broader areas such as role functioning or quality of life (QoL). Despite calls for a broader approach to outcomes [8-10], a recent review article on treatment outcome assessment listed no measures other than those of symptoms and behaviors and did not use the term 'quality of life' [11].

The impact of EDs on broader life functioning is well documented [12], and measures of treatment success that reflect these broader areas are in keeping with the trend in contemporary health services toward measuring outcomes, such as QoL, that are important to patients [13]. In practice, the use of broader outcome measures in EDs has been limited by a lack of availability of specific QoL measures that are suitable for a broad age range (young adolescents through mid-life adults).

Generic measures such as the NHP, SF-36/12, and the WHOQoL-Bref have been used in ED samples in research studies and have been found to discriminate between normal and ED populations in measured functioning or QoL [12], but they have some drawbacks. Some domains and items may be insensitive for some ED diagnoses [14], they are not developmentally oriented in content or language, and responsiveness may be inadequate for evaluative purposes [15,16]. Adult QoL measures are usually not appropriate for use in children and adolescents [17]. Wording and interpretation problems with the SF-36 have been found for some patient groups including EDs [14,18,19].

Many authors have emphasized the importance of measuring QoL in a way that is meaningful to patients in health services including ED services [13,20-24]. Meaningful measurement requires more than trivial involvement of patients in instrument development. Such involvement is infrequent in measure development, especially in younger ages. In a recent systematic review, Cremeens et al. found that a *minority *of instruments specifically developed for children included them in development, typically relying instead on expert panels to generate items [25].

There has been increasing consensus in the ED field that a specific, relevant and responsive QoL measure is needed to evaluate patient outcomes, improve ED programs, and test new treatments [12,19,26-29]. Three new disease-specific instruments for EDs were reported in 2006 [27-29]. Two of these focus predominantly on symptoms and behaviors [28,29]; one was tested on inpatients only [29]; one addresses four broader domains but has no ED symptom and behavior items [27], and none reported considering suitability for adolescents in design. This research has rectified a total lack of knowledge about disease-specific QoL measurement for EDs in a short time, yet some challenges remain. de la Rie et al. recently studied patient preferences for QoL domains using a generic individualized QoL tool and these preferences are not in alignment with the domains of existing measures [23]. Neither does any of the existing measures allow for assessment of individual QoL, which is essential to client centered care [20,23].

An additional concern which has been raised but not yet addressed is the influence of ego-syntonicity on self-reports of QoL [12,14,26]. Ego-syntonicity occurs when the illness behaviors are initially consonant with the individual's desires and goals (e.g. he/she may feel in control and proud of achieving a desire weight loss and may have received compliments on that) [30]. He/she will deny or will be unable to comprehend the negative effects of the behavior. In the context of QoL measurement, this lack of insight could manifest as inflated QoL reporting (typically early in treatment) despite clear objective evidence of adverse effects on health and functioning.

This article reports on the development and validation of a condition-specific QoL instrument (the Eating Disorders Quality of Life Scale (EDQLS)), designed to minimize response bias attributable to ego-syntonicity in EDs, to allow for both standard and individualized QoL assessment, to be sensitive to change with treatment, and to be developmentally appropriate for adolescents and young adults, while also being suitable for adults with EDs. In this article, we report on findings arising from the analysis of baseline data from a longitudinal multi-site development and validation study.

## Methods

### The general approach

The development of the EDQLS was grounded in the World Health Organization's definition of QoL, which conceptualizes QoL as subjective, multi-dimensional, having positive and negative aspects and covering at a minimum physical, psychological and social dimensions. It is *"individuals' perception of their position in life in the context of the culture and values system in which they live and in relation to their goals, expectations, standards and concerns" *[31]. This definition is congruent with draft industry guidelines for "health-related quality of life", which, in the context of medical treatment, is the patient's perception of the impact of the illness and its treatment on, at a minimum, these same domains. [32]

The EDQLS was developed with an *evaluative *purpose, that is to measure change over time *within *individuals, versus for example, a *discriminative *measure – which is primarily intended to show differences between patient groups or patients and healthy individuals [33,34]. Thus the emphasis in design was on responsiveness, for both assessment of patients' treatment progress and the outcomes of new treatments such as in clinical trials. EDQLS content was selected to measure the broader aspects of life that were confirmed to be important to patients *including *those which are specifically affected by EDs and their treatment (i.e. health-related QoL). However, too much overlap in content with existing instruments that measure ED symptoms and behaviors alone was avoided.

EDQLS development was guided by published standards [22,24,34,35], with emphasis on six recommendations: to define QoL at the outset, to specify the intended purpose, to use multiple sources for item generation (especially patients), to reduce items according to respondent importance ratings and meaningfulness/sensibility rather than relying solely on factor analytic processes, to use systematic approaches to pre-testing, and to incorporate longitudinal validation for evaluative instruments.

Three disorder-related design issues were addressed. First, the potential for underreporting of sensitive life issues related to QoL in EDs (e.g. substance abuse, sexual abuse) in in-person interviews at the domain/item development stage was considered. To address this issue a set of internet-based narratives written and posted anonymously by individuals with current or past EDs was included. Second, concerns about potential reporting bias due to the egosyntonic nature of EDs (in particular AN) were attended. Third, a tool suitable for the age range that encompasses most presenting patients was desired, to reduce the necessity for multiple instruments. Two measurement-related design issues were also addressed. First, standardization for comparability was valued but not completely at the expense of allowance for assessment of individual QoL preferences. Second, the need to systematically select items that were amenable to change (in keeping with the evaluative purpose) was emphasized in design.

### Development of the EDQLS – domain and item generation

**Figure 1:**
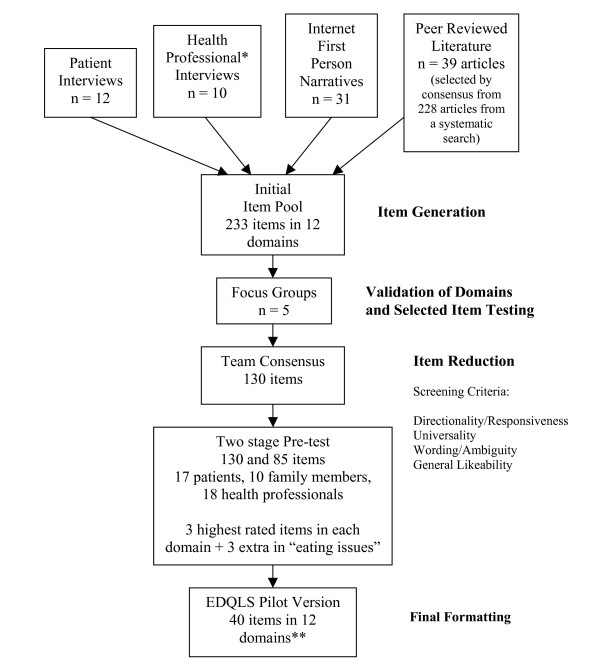
**The EDQLS Development Process**. * included nursing, dietetics, pediatrics, psychology, psychiatry, social work and family medicine; all with EDs clinical experience. ** Final domains: cognitive functioning, education/vocation, family and close relationships, relationships with others, future/outlook, appearance, leisure, psychological health, emotional health, values and beliefs, physical health and eating issues.

A multi-source and multi-stage process (Figure 1) with participation of adolescents and young adults with EDs, their families and treatment providers was used to develop and finalize the draft instrument. Participants in the development stage were patients with diagnosed eating disorders (AN, BN and EDNOS) from the Calgary Eating Disorders Program (CEDP), aged 14 and over, and their family members. It was recognized that special, in-depth methods (e.g. cognitive interviewing) would be needed to confirm suitability of the EDQLS for those under age 14, and numbers of younger eligible participants were very small, so validation in this group was set-aside for a separate study. The CEDP provides day and outpatient treatment and serves a regional population of nearly 1 million. Illness severity is quite high due to high service demand and limited availability. Health professionals with ED experience from five other service sites across Canada were also involved in development.

Four sources of material were tapped for domain and item generation (the peer-reviewed literature, treatment provider interviews, patient/client interviews and internet-based first person narratives).

First, a systematic literature search for abstracts on the topic of QoL in EDs was conducted, yielding 228 abstracts. Five investigative team members (both researchers and clinicians) then independently rated each abstract on a standard relevancy statement "*the experience or phenomenon of eating disorders and its impact broadly on the person's life from the patient's perspective is the central focus of the abstract" *using a four-point scale. Articles that were rated as relevant by at least three raters and had scores above an a priori cut-off were retained for content analysis (N = 39).

Second, 10 health professionals with clinical experience in EDs from five sites and multiple disciplines (nursing, dietetics, pediatrics, psychology, psychiatry, social work and family medicine) were interviewed.

Third, in-depth semi-structured interviews were held with 12 patients ranging in age from 16 to 29 (58% under 18). The number of participants involved in this phase was constrained by the number of eligible patients in the CEDP, the challenges of recruitment in this patient population and the recognition of the need for participants in subsequent phases of testing. The questions used to elicit thoughts on ED and QoL in patient and provider interviews were similar to the operational definitions for extracting concepts about ED and QoL from articles and narratives. These questions targeted phrases or ideas that addressed the "areas of life most affected by EDs" and those "most impacted by recovery". Approximately 2000 units of text were extracted from the four sources using content analysis [36]. After eliminating redundancies, five team members independently grouped the concepts using a card-sort approach, and consensus discussion resulted in 12 QoL domains. Next, 233 item stems were derived from the text units for each domain, retaining the patient's phrasing where possible.

Finally, 31 first person narratives posted by individuals with EDs on the internet and reporting on impact of these illnesses on QoL were systematically sampled and similarly analyzed. The first person narratives were used to confirm themes arising from patient interviews, to identify sensitive issues that might be under-reported in face-to-face interviews, and to improve generalizability of extracted themes beyond the local site (see Adair et al. 2006 for more detail on this component) [37]. The addition of this component increased the material representing the patient perspective to 43 individuals.

### Development of the EDQLS – item reduction

Item generation was followed by four stages of pre-testing to reduce the item pool. In the first step, the 12 domains of QoL as well as a selection of 59 items with specific concerns were tested in two in-depth focus groups with five patients (aged 15–22). For example, participants were probed about whether the item captured their language and the language of their peers and whether they felt it was relevant to their QoL. The domains were endorsed as presented and suggestions for item additions and revisions were made. Next the item pool was reduced to 130 through a process of investigative team ratings on four principles, followed by team consensus decisions. The principles were:

• ***Directionality/Responsiveness*: **the item is expected to be sensitive to change in a linear direction over time with treatment (at this stage a specific assessment of the risk of response bias for each item attributable to egosyntonicity was also made)

• ***Universality: ***the item captures behaviors/feelings of individuals across ED diagnostic groups and a broad age range with particular attention to inclusion of younger ages

• ***Wording/Ambiguity: ***the item is clearly worded and understood and is unlikely to evoke a variety of interpretations

• ***General Likeability: ***the item resonated with focus group participants and is felt to be appropriate for the target population

At this stage the items were also mapped against those in the Beck Depression Inventory (BDI-II) [38] and the Eating Disorders Inventory (EDI-2) [39] and the degree of conceptual overlap was rated by the investigative team to be reasonably minimal (12 and 20% similar items respectively). Next the 130 items were pre-tested with 17 patients (aged 14–40), 10 family members and 18 health professionals. At this stage each item was rated as "good" or "not good" according to its relevance for QoL and its ability to show change with treatment. Respondents were then asked to identify the "best three" items in each domain, and to provide general comments about wording (clarity, comprehension, ambiguity). Responses were collected on a self-completed pre-tested form with research assistant support as needed. A straightforward arithmetic algorithm was used to identify the highest ranked items and items were edited according to specific suggestions. Ratings were consistent across respondent groups, but where they diverged decisions to retain or eliminate items followed the preference of the patients. After consensus discussion, the investigative team retained the *three *highest rated items overall in each domain.

The final domains are listed at the bottom of Figure 1. The *six *highest rated items in the eating issues domain were retained to ensure specificity of the instrument to EDs, resulting in a final set of 39 items across 12 QoL domains. At the pilot stage we included one additional item with nearly identical wording and meaning to another as a specific test of internal consistency. Final formatting of the EDQLS included response scaling with a 5-point scale with anchors "strongly disagree", "disagree", "neither agree or disagree", "agree", and "strongly agree". Options for response scale anchor terms had also been tested with patients and there was a strong preference expressed for the endorsement type anchors (i.e. "strongly disagree" through "strongly agree") over frequency type response anchors (e.g. always, sometimes, never). Participants reported great difficulty with recall on proposed frequency anchors and commented that they were "just guessing". Items were also subjected to a readability check (Flesch-Kincaid grades 5–7) and were balanced for polarity (negative vs. positive wording). Inclusion of both item types was intended to minimize the effects of response sets. This necessitates a reverse scoring procedure before analysis. Example items from the final 40-item EDQLS are *"I have a lot of rules about food" *(eating issues domain) and *"I feel connected to others" *(relationships with others domain). A single item global QoL rating (on a 10 point rating scale) was added for overall construct validity assessment [24]. To allow for a more individualized assessment a *separate *section of the instrument was designed which lists the 12 QoL domains and permits respondents to rate the importance of each as well as up to two additional self-nominated domains on a five-point importance scale. These importance ratings are not used to weight the total domain scores derived from the core 40 items as per current recommendations [40] but they provide an opportunity for the patient and clinician to consider and address unique QoL issues and goals as an adjunct to the standard scores.

### Pilot and multi-site samples

Females and males over age 14 with a clinically confirmed ED diagnosis (AN, BN or EDNOS) were eligible for both the pilot and multi-site field test. Pilot participants came from the CHR EDP and no males participated. The only information collected other than the EDQLS in the pilot was age, sex, time in treatment, and completion time. For the multi-site study, 12 programs (two in Nova Scotia, three in Manitoba, five in British Columbia, and two in Alberta) providing any of inpatient, outpatient, day treatment and/or consultation to adolescent or adult patients participated.

### Validation measures and other variables

Validation instruments included the Short-Form-12 (SF-12) [41], the Quality of Life Inventory (QoLI) [42] and the 16D [43]. The SF-12 is a brief version of the SF-36, an extensively tested and validated health status instrument used in many patient populations to measure health-related functioning and frequently used as an indicator of QoL. It has 12 items that address activities such as playing golf and climbing stairs, plus limitations in performing physical tasks, and in working or socializing due to physical and emotional problems or pain and provides summary scores for mental and physical functioning/status [41]. The QoLI is a generic QoL life instrument with 32 items addressing 16 areas of life (health, self-esteem, goals and values, money, work, play, learning, creativity, helping, love, friends, children, relatives, home, neighborhood and community) and includes importance and satisfaction ratings for each. It has been validated in several clinical and non-clinical populations with internal consistency values ranging from .77 to .89 [42]. The 16D is a generic QoL measure designed specifically for youth aged 12 to 15. It has a single item in each of 16 domains (mobility, vision, hearing, breathing, sleeping, eating, elimination, speech, mental function, discomfort and symptoms, school and hobbies, friends, physical appearance, depression, distress and vitality) with good test-retest reliability and known group validity [43]. The 16D was chosen to assess the appropriateness of the EDQLS for adolescents.

Baseline severity of illness, psychiatric comorbidity and stage of readiness to change ED behaviors were hypothesized as key predictors of QoL and potential confounders of other group comparisons. Standardized and validated instruments – the Brief Symptom Inventory (BSI) [44], EDI-2 [39] and the Motivational Stages of Change for Adolescents Recovering from an Eating Disorder (MSCARED) were used to measure these variables respectively [45]. The MSCARED is a self-report measure to assess stage of readiness to "take action" against an ED behavior. The respondent endorses one of six statements, each representing a stage of change. In an ED sample, good test re-test reliability (r = .92), concurrent validity with clinician ratings (r = .79) and predictive validity with treatment outcomes [45] were found for the MSCARED. The battery of measures was reviewed by clinical collaborators at the sites for appropriateness for the target population. The content of standard instruments could not be changed, but this step was felt to be important for identifying any issues with items to inform interpretation of results. The instrument battery was also pre-tested with eight adolescents/young adults aged 13 to 31 for burden, comprehension, and completion time.

Other variables of interest: age, sex and rating of state wellness (current day) were collected via self-report during baseline instrument administration. The remaining variables (site, treatment status at enrolment (inpatient or outpatient)), psychiatric and medical comorbidity, prior treatment, age at first symptoms, illness duration, current program treatment duration and most recent BMI were collected from the health record using a standard, pre-tested abstraction form.

### Data collection and management

Participants were recruited through presentations by the research assistant in group therapy sessions, and by individual clinician referrals. The baseline battery of instruments was self-completed in hard copy on-site with assistance as needed, then taken back to the central study office. There, data were entered to an SPSS database and error rates were measured on a 10% random sample and found to be below 1%. Missing data was minimal, and handled using standard decision-rules (e.g. inserting subscale means) and dual-rater agreement on items requiring judgment (such as potentially ambiguous respondent corrections).

### Analysis

Cronbach's alpha was computed for internal consistency reliability of the total scale and each developed domain. Next, corrected item to total correlations and the impact of item deletion on Cronbach's alpha were evaluated. There is a lack of consensus on whether stability over time (measured by test re-test reliability) is a suitable psychometric characteristic of an evaluative instrument. By design the scores of an evaluative instrument should change over time in response to treatment, making responsiveness the more important characteristic [35]. For this reason we deferred examination of test re-test reliability to a subsequent study. Construct/criterion validity was examined in bivariate analysis of EDQLS total scores according to demographic and clinical characteristics using ANOVA and Tukey's HSD post-hoc analysis. A priori hypotheses for convergent and divergent correlations between EDQLS items and total score and criterion instrument items/total scores were tested using Spearman correlations and Pearson's correlations. This analysis focused on items and total scores because of the preliminary nature of the domains. Principal components analysis (PCA) and item response theory (IRT) analyses were used for initial, exploratory review of instrument and item properties after confirmation of data suitability [46]. Next an initial impression of the number of underlying factors was made using Kaiser's criterion (eigenvalues >= 1), Scree plots and Horn's parallel analysis using Monte Carlo PCA [47], followed by varimax oblique rotations on item clusters. Muraki and Samejima graded response models (in Parscale software) were used for exploratory IRT analysis [48].

### Ethics

All stages of the study were reviewed and approved by the Conjoint Health Research Ethics Board at the University of Calgary. The protocol for the multi-site validation study was approved by the respective committees for each jurisdiction.

## Results

The pilot sample comprised 41 females with EDs from the Calgary program aged 15–44 (mean 24.4, SD 8). Seventeen (43%) had been in treatment for less than three months; 11 (28%) for 3–8 months, and 12 (30%) for more than eight months. Internal consistency of the EDQLS was high in the pilot (Cronbach's alpha = .95). Exploratory results of the pilot sample were so similar to the baseline data for the multi-site study that the samples were pooled for the item analysis reported herein (N = 171). Subgroup and comparative instrument findings are reported for the 130 participants from the multi-site study.

Participant demographic and clinical characteristics are shown in Table 1. Only six males participated due to small proportions of males in all participating programs. Nearly 30% of the sample was under 18 years of age and three quarters was under 29 years.

**Table 1:** Participant demographic and clinical characteristics (baseline)

**Characteristic**	**N**	**Mean (SD) Range**
Age (years)	170	25.3 (10) 14–60
Age at First Symptoms (years)	123	15.3 (4.7) 8–40
Duration of Illness (years)	122	9.7 (9.1) 0–45
Time in Treatment (months)	129	12.5 (15.8) 0–85
Body Mass Index (weight in kg/height m^2^)	121	20.6 (4.5) 14–39
	**N**	**Frequency (%)**

Sex (% female)	171	165 (96.5)
Treatment Status at Enrolment (% outpatient)	171	160 (93.6)
Time in Treatment (current service)	129	35 (27.1)
<3 months		52 (40.3)
3–12 months		42 (32.6)
12+ months		86 (66.2)
Previous Treatment (%)	130	48 (28.1)
Primary Diagnosis	169	
AN (restricting type)		24 (14.0)
AN (binge-purge type)		55 (32.2)
BN		42 (24.6)
EDNOS		
Psychiatric comorbidity (% >1 DSM-IV diagnosis)	130	89 (68.5)
Medical comorbidity (% any)	130	45 (34.8)
Body Mass Index Category	121	
Underweight		42 (34.7)
Normal		65 (53.7)
Overweight		10 (8.3)
Obese		4 (3.3)
Stage of Change (MSCARED category)	130	
Pre-contemplation		1 (.8)
Contemplation		10 (7.7)
Preparation		20 (15.4)
Action		65 (50.0)
Maintenance		32 (24.6)
Recovery		2 (1.5)

### Item distributions, overall score and reliability

Mean completion time was five minutes (range 2 to 11). All items showed good dispersion (minimal ceiling or floor effects) and all items had responses in all categories. The total EDQLS raw score is derived by summing the item ratings. The mean total score was 110 (SD 27.6; range 56 to 187) out of a total possible score of 200 (representing the highest QoL). Subscale scores are also sums of item ratings for the 12 theoretically developed domains. The scale was found to be highly internally consistent (Cronbach's alpha .96). This value was virtually unchanged for any item deletion. Item to total correlations ranged from .28–.76 with only three items having item to total correlations below .40 and no items completely redundant. Cronbach's alphas between defined domains and the overall score were as follows: cognitive functioning (.73); education/vocation (.76); family and close relationships (.36), relationships with others (.69), future/outlook (.64), appearance (.76), leisure (.50), psychological health (.71), emotional health (.68), values and beliefs (.72), physical health (.61) and eating issues (.79). Two of the poorest fitting items were from the family and close relationships domain and one from the leisure domain.

### Validity

**Figure 2:**
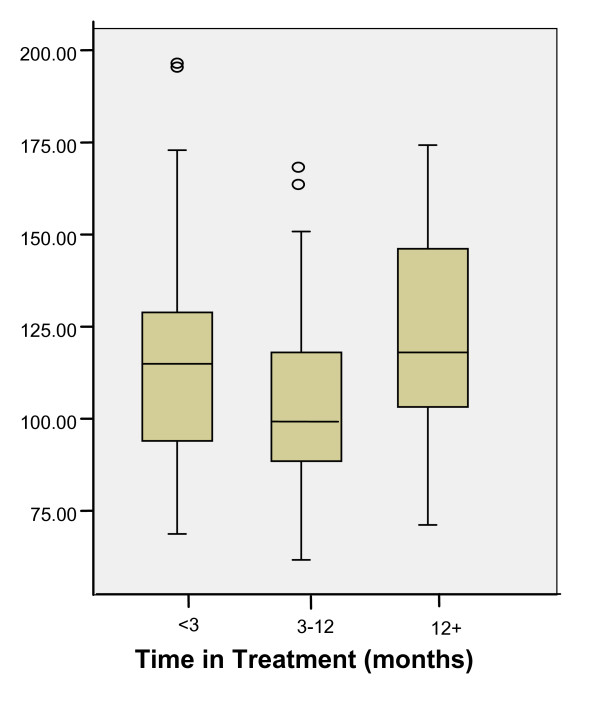
EDQLS Total Scale Scores by Time in Current Treatment Program.

**Figure 3:**
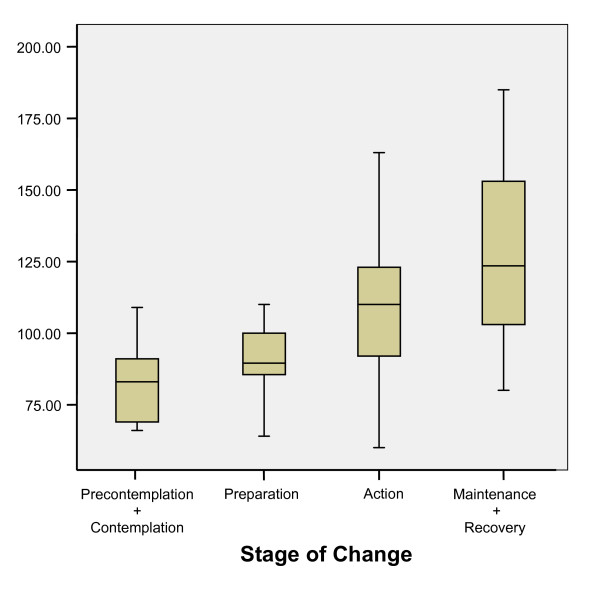
EDQLS Total Scale Scores by Stage of Change Reported at Baseline.

Patterns of EDQLS scores by other variables are shown in Table 2. EDQLS score distributions varied somewhat by program and province but these differences were not statistically significant (F 1.34, p = .20 and F = .802, p = .49 respectively). Scores were lower for inpatient vs. outpatient and males vs. females but these differences were not tested for statistical significance because of very small numbers of males and inpatients. Patterns of reported QoL across all variables were consistent with predicted directions. Differences were not significant by age, diagnosis, medical comorbidity, BMI or age at first symptoms. Those in treatment longer reported statistically higher QoL scores except for the initial group (in treatment less than 3 months) (Figure 2). Those with psychiatric comorbidity (greater than one DSM IV diagnosis) had significantly lower EDQLS scores, as did those with higher levels of psychiatric symptom severity (on the BSI) and ED symptom severity (on the EDI-2). The severity patterns held across all nine subscales of the BSI and all 11 subscales of the EDI-2 at the p < .001 level. A strong linear and statistically significant association was found with reported stage of change – with a 43 point spread in EDQLS scores between those in pre-contemplation or contemplation and those in recovery or maintenance (Figure 3).

**Table 2:** Relationships between EDQLS Scores, Demographic and Clinical Variables

**Variable**	**N**	EDQLS Total Scale Score* Mean (SD)	**Test Statistic and significance level**†
Age Group			
< 18	50	115.7 (30.5)	F = 1.31 ; .273
19–24	55	107.9 (27.8)	
25–29	21	102.8 (30.3)	
>30	44	109.5 (21.4)	
Primary Diagnosis			
AN (restricting type)	48	108.6 (28.3)	F = .785 ; .504
AN (binge-purge type)	24	105.2 (28.0)	
BN	55	109.6 (24.0)	
EDNOS	42	115.2 (31.1)	
Psychiatric Comorbidity			**F = 9.10 ; .003**
Yes	88	103.3 (22.5)	
No	42	117.9 (31.3)	
Medical Comorbidity			
Yes	45	102.9 (22.0)	F = 2.63 ;.107
No	85	110.8 (28.2)	
BMI Category			
<18	42	110.4 (30.3)	F = 1.22 ; .304
18.5–24.9	65	109.0 (25.9)	
25–29.9	10	97.3 (18.9)	
30+	4	90.7 (15.6)	
Age First Symptoms			
<12 years	32	104.0 (22.3)	F = 1.75 ; .161
13–15	49	114.2 (25.7)	
16–18	20	112.7 (35.7)	
18 or over	22	101.6 (22.3)	
Stage of Change**			
Pre-contemplation/Contemplation	11	81.9 (15.0)	**F = 15.1 ; .000**
Preparation	20	90.8 (12.3)	
Action	65	108.7 (22.3)	
Maintenance/Recovery	34	125.4 (29.6)	
Time in Treatment			
<3 months	35	110.3 (29.0)	**F = 4.65 ; .011**
3–12 months	52	100.0 (23.6)	
12 months or more	42	116.0 (25.4)	
Psychiatric Symptom Severity^			
Low	43	135.3 (22.6)	**F = 86.9 ; .000**
Med	45	101.3 (11.2)	
High	42	87.4 (16.4)	
ED Symptom Severity#			
Low	43	136.1 (21.2)	**F = 95.3 ; .000**
Med	42	100.5 (12.5)	
High	45	88.1 (26.4)	

* EDQLS – possible scores range from 0 to 200 with higher scores indicating higher QoL.

** As measured on the MSCARED

^ BSI – Global Severity Index

# EDI-2 – Total score across all subscales

† using Analysis of Variance. Differences that are statistically significant at least at the .01 level are in bold text.

Findings for comparisons with criterion QoL instrument items and scale scores (including convergent and divergent validity) are shown in Table 3. The magnitudes of correlations were moderate to strong for nearly all comparisons predicted to be correlated. The mean correlations across items predicted to be correlated were rho= .42, .37 and .55 for the SF-12, QoLI and 16D whereas the mean correlation for items predicted to be poorly correlated was rho= .19). Overall the EDQLS total score was highly correlated with the 16D weighted total score (r =.78, p < .001), the QoLI weighted total score (r = .61, p < .001) and the mental subscale of the SF-12 (r = .71, p < .001). A lower correlation, as expected, was found between the EDQLS total score and the SF-12 physical subscale (r = .37, p < .001). Construct validity was also supported by highly significant positive correlations with the EDQLS global quality of life item, the SF-12 health status item and the current wellness question.

**Table 3:** EDQLS Total Score and Item Correlations with Validation Instruments for 130 Multi-site Participants at Baseline

**Convergent Validity EDQLS Item**	**Criterion Item – SF12**	Spearman's rho*	**p**
3 I have an open honest relationship with someone outside my family	12 Interference with social activities	.22	.01
10 I feel connected to others	12 Interference with social activities	.40	.000
16 I have fights with my family members about food and eating *	12 Interference with social activities	.33	.000
19 I turn down opportunities to go out with friends *	12 Interference with social activities	.62	.000
22 People don't understand me *	12 Interference with social activities	.37	.000
28 I have to be in a relationship to feel good about myself *	12 Interference with social activities	.01	NS
6 I hardly ever worry	9 How much ..time...felt calm/peaceful	-.55	.000
21 I feel hopeful about the future	11 How much ..time....felt downhearted	.51	.000
9 I have lots of energy	10 How much.. time ...did you have lots of energy	-.75	.000
**EDQLS Item**	**Criterion Item – QoLI**		

11 I get satisfaction from my main activity	10 Satisfaction with work	.55	.000
28 I have to be in a relationship to feel good about myself *	19 Importance of love	-.28	.001
28 I have to be in a relationship to feel good about myself *	20 Satisfaction with love	.06	NS
3 I have an open honest relationship with someone outside my family	19 Importance of love	.07	NS
3 I have an open honest relationship with someone outside my family	20 Satisfaction with love	.38	.000
19 I turn down opportunities to go out with friends *	22 Satisfaction with friends	.30	.001
16 I have fights with my family members about food and eating *	26 Satisfaction with relatives	.17	NS
21 I feel hopeful about the future	6 Satisfaction with goals and values	.52	.000
27 I think about what I would like to do in my life	6 Satisfaction with goals and values	.38	.000
1 I have fun with others	12 Satisfaction with play	.33	.000
14 I enjoy relaxing in my free time	12 Satisfaction with play	.28	.001
24 I enjoy participating in different activities, not just exercise	12 Satisfaction with play	.27	.002
7 I show my true self to others	4 Satisfaction with self-esteem	.39	.000
26 I am able to see good qualities in myself	4 Satisfaction with self-esteem	.60	.000
30 I put myself down a lot *	4 Satisfaction with self-esteem	.68	.000
5 My health is more important than my physical appearance	1 Importance of health	.41	.000
31 I feel self-conscious about my body around others *	4 Satisfaction with self-esteem	.60	.000
**EDQLS Item**	**Criterion Item – 16D**		

4 I have trouble concentrating	14 Mental function (thinking clearly and logically)	-.52	.000
20 I can focus on things other than food	14 Mental function (thinking clearly and logically)	-.39	.000
29 Thoughts about food and eating dominate my life *	14 Mental function (thinking clearly and logically)	-.41	.000
39 The eating disorder has taken over my life *	11 School and hobbies	-.59	.000
19 I turn down opportunities to go out with friends *	13 Making friends or being with them	-.45	.000
13 I see positive things in my appearance	10 Weight, height and what I look like	-.61	.000
9 I have lots of energy	1 Feeling healthy and energetic	-.67	.000
25 I'm constantly trying to fix my body *	10 Weight, height and what I look like	-.75	.000
38 I'm obsessed with my weight or my body shape *	10 Weight, height and what I look like	-.75	.000
24 I enjoy participating in different activities, not just exercise	11 School and hobbies	-.35	.000
31 I feel self-conscious about my body around others *	10 Weight, height and what I look like	-.68	.000
21 I feel hopeful about the future	16 Feeling sad, melancholic or depressed	-.49	.000
6 I hardly ever worry	4 Feeling anxious, stressed or nervous	-.55	.000
32 My sleep is restful	6 Problems with sleeping	-.51	.000
	**Criterion Item or Scale Score**	**Pearson's r**	**p**

**EDQLS Total Score**	SF-12 Health status item	-.48	.000
	Please rate how well you feel today.	.56	.000
	Please rate your overall quality of life in the last week (EDQLS global QoL item)	.73	.000
	SF 12 Mental Subscale	.71	.000
	SF 12 Physical Subscale	.37	.000
	16D Weighted Total Score	.78	.000
	QoLI Weighted Total Score	.61	.000
**Divergent Validity EDQLS Total Score**			
	16D 2 Vision	-.08	NS
	16D 3 Breathing	-.32	.000
	16D 5 Hearing	-.21	NS
	16D 9 Speech	-.28	.001
	16D 12 Walking	-.04	NS

* refers to items that are reverse scored for analysis.

Despite a relatively small sample size for PCA and IRT analyses the initial tests of sample suitability were reassuring. Bartlett's test of sphericity was highly significant (p < .001) and the Kaiser-Meyer-Olkin value was .93. Most of the values in the item correlation matrix fell between .3 and .6. PCA indicated up to eight factors accounting for 64.4% of the variance using the eigenvalue >1.00 criterion with the first two components accounting for 44.1%. The scree plot and parallel analysis suggested two to three factors. Varimax oblique rotation provided an initial sense of possible item groupings with one cluster indicative of eating disorders behaviors and effects, and others suggestive of future outlook, work/leisure and psycho-social-emotional issues. However, thematic interpretation was deferred for confirmatory analysis in larger samples. Despite the relatively small sample, both IRT models converged satisfactorily. The Samejima model, which does not assume equal intervals between response options across items fit the data significantly better than the Muraki model, which does (χ^2^= 333, p < .05). Item characteristic and item response curves indicated large concerns with only one item in both models and moderate concerns with 2–7 items depending on the model. The two items of greatest concern are those identified earlier in the classical analysis and point to a need for item replacement in the family and close relationships domain, if this pattern holds in the longitudinal analysis.

## Discussion

A condition-specific QoL scale for EDs has been developed, for which initial results are promising. Face and content validity are supported by a multi-source, patient-centered development process. Results suggest that an overall construct of QoL is being tapped by the EDQLS, with a primary domain of eating disorders issues, and some additional item clusters representing broader life issues. Only a few items display poor fit characteristics.

Validity is supported by moderate to strong correlations for most hypothesized relationships with well constructed and validated generic QoL instruments. Appropriateness for adolescents was supported by the pattern of correlations with the 16D and correlations in total scores with the SF-12 and QoLI offer reassurance that the adolescent perspective was not taken at the expense of appropriateness for adults. A much stronger correlation between the EDQLS total score and the SF-12 mental component vs. the SF-12 physical component is consistent with previous studies [14,19,49,50]. It underscores the strong psychosocial pathology of EDs but also suggests that the physical functioning items of the SF-36/12 may not be optimal for capturing the impact of EDs on physical health, and is congruent with concerns about suitability of some of the SF-12 items for the ED population [14,19]. Several of our participants spontaneously questioned the suitability of the SF-12 in response to its generic nature (e.g. "*some of my experiences of an eating disorder weren't covered much*") and some noted that examples provided for some SF-12 items (e.g. bowling or playing golf) were difficult to relate to.

Patterns of EDQLS scores were consistent with expectations although power was not adequate for some analyses. No statistically significant differences were found by age, age at first symptoms, medical comorbidity, BMI or diagnosis. In a community sample, Hay et al. found no association between age of onset and QoL in BN although older individuals reported lower QoL [51]. Lower QoL has also been reported for older individuals in other psychiatric disorders [26] but in the QoL literature in ED age is often used as an adjusting variable – rather than being reported separately. Differences in QoL according to BMI have been examined for diagnostic subpopulations only (e.g. Hay 2003) [51] and typically show lower QoL at low and high BMI levels. Our results for BMI are consistent with that pattern, but were not statistically significant.

As expected, those with comorbid psychiatric diagnoses and higher levels of psychiatric and ED symptom severity reported significantly lower QoL as measured by the EDQLS, a finding that is firmly established in the QoL in ED literature [9,12,19,27-29]. It was also encouraging that, with the exception of the most recent admission group, participants who were in treatment longer reported significantly higher EDQLS scores. This finding suggests that treatment is associated with improved QoL and that the EDQLS may be responsive to detecting changes that occur over time. In this cross-sectional analysis, confounding by age and severity would likely dampen this association since those in treatment longer would tend to have greater severity of illness and would be older. Longitudinal analysis will allow disentangling of these factors and provide stronger evidence for responsiveness. A highly statistically significant association was found between EDQLS scores and sequential levels of reported stage of change. This suggests that QoL improves with readiness to change, and by implication, a treatment approach which considers stages of change theory is consistent with a recovery process that results in better QoL.

With respect to diagnosis – several studies have found no differences among ED diagnostic groups on QoL as measured by the SF-36/12 [9,49] but Mond et al. did find that the AN restricting subgroup reported significantly better QoL than other patient groups after controlling for levels of general psychological distress [26]. Doll (2005) had similar findings but also noted that those with AN also reported more depression, self-harming behavior and suicidal ideation [14]. These authors' explanation for the finding was that the SF-36 is insensitive in measurement of the way that distress in AN impacts functioning. The finding in the current study of no difference by diagnosis was a desirable endpoint in that items were deliberately and systematically selected to apply across diagnoses and to minimize egosyntonic responding. The perspectives of health professionals and families were also incorporated and participants at later stages of recovery were asked how they might have responded to specific items earlier in treatment.

These efforts seemed to minimize this bias on a group basis, but the phenomenon may still have been operating in some respondents. A handful of scores above 180 on a scale of 200 seemed to be unrealistically high for individuals referred to tertiary health services as a result of significant symptom levels. These scores, informally, seemed to be reported by individuals who were younger and had a diagnosis of AN. One was an inpatient, several participants were underweight (as measured by their BMI), and others were recent entries to treatment with shorter durations of illness. It may be that the ED had not yet fully impacted the broader life of these individuals, but it is also possible that these patients lacked insight into the impact of their illness on their QoL as a result of egosyntonicity. Further study of these initial observations is needed. Until more is known about the reasons for this phenomenon it may be prudent to use repeated measures at several time points in treatment (vs. start and endpoint measures) and some caution should be taken in interpreting QoL scores in early treatment stages. Our data suggest that after about three months of treatment score trajectories appear to be valid, and stronger evidence is forthcoming from our longitudinal study. While a central principle of QoL measurement is that it must be reported by the patient him or herself, caution is recommended in using scores based on self-reports that are in great divergence with the observations of clinicians or family members to make important decisions about treatment outcome. Stage of change ratings may also be valuable adjunct information.

Twelve domains have been endorsed by our respondents as being important to their QoL; which is a large number in comparison with the number of domains in the other new disease-specific instruments which range from four to eight [27-29]. However, using similar patient-centered processes, de la Rie and colleagues identified 11 domains of importance (sense of belonging, family/friends, self-image, well-being, health, ED psychopathology, life-skills, work/education, sense of purpose/meaning, financial/living condition) most of which parallel the EDQLS domains quite nicely. Twenty-seven respondents in this study also nominated individually relevant domains, confirming the utility of including an individualized approach, but most of these were specific instances of one of the existing domains or relatively unique individual values (music, religious faith, relationship with nature). The financial domain identified by both Engel and de la Rie [23,27] did not emerge in the current study – possibly because of a combination of younger participants who are not yet financially independent and the context of publicly funded healthcare services in Canada.

General response to the EDQLS by participants in our study was very positive. Some respondents commented that it was refreshing and interesting to be completing a scale that addressed their broader life interests and concerns, not just ED symptoms and behaviors. One respondent commented *"As eating disorders are about more than food/weight, I think that this type of study/questionnaire is a more accurate representation of where a person is at in regards to the effects of an ED"*. The contribution that adolescents and young adults can make to instrument development may be underestimated in QoL research. In this research, young participants were insightful about their QoL, able to capably articulate their ideas about the utility of items, and able to offer excellent suggestions for revision. It was humbling to find, that some of the most favored items of the "expert" investigative team did not resonate with patients. Completion of the EDQLS was reported to be easy and took only about five minutes, which makes the instrument acceptable and practical for administration in clinical settings.

For the first time there is a range of choices for disease-specific QoL measurement in the ED population [27-29]. Instrument choice depends on purpose and population. The EDQLS offers comprehensive measurement of broader quality of life domains that have been confirmed to be important to patients as well as a symptom and behavior domain. It has been validated against well established generic QoL measures, predicts severity levels on symptom measures, and is suitable for the age range of most current ED patients.

Some limitations apply to this work. First, the development work involved patients from only one program; however the patient perspectives from the internet were geographically broad, health professional perspectives spanned multiple disciplines and five clinics, and the validation study included patients across 12 programs in four provinces. Domains were developed through consensus processes to ensure broad coverage of QoL in this population, however, it is recognized that this number of defined domains may never align fully with factor analysis output. Sample size for baseline data analysis was not sufficient for strong conclusions about differences for some variables and for confirmatory factor and IRT analyses. These will require larger samples. The EDQLS has been tested in only six males so cannot be considered to be sufficiently validated for boys and men. Suitability for diverse ethnic groups is unknown.

French and Spanish translations have been produced but are not yet validated. The EDQLS can not be recommended for use in patients younger than 14 without further validation although the reading level and initial clinical impressions suggest that the suitable age boundary may be lower. Egosyntonic responding was considered in EDQLS development but may not be completely eliminated for some respondents. The extent to which socially desirable responding may be influencing EDQLS scores remains to be assessed. Proxy versions for health professionals and family members have been developed but not yet tested. Clinically meaningful cut-points and differences have yet to be specified for the instrument and responsiveness to be confirmed.

## Conclusion

The EDQLS appears to be a promising condition-specific QoL instrument that is appropriate for ED patients as young as 14 and as old as 60. The findings that those in treatment longer and in later stages of change report higher QoL are encouraging and suggest that QoL outcomes responsive to treatment may be measurable by the EDQLS. Further research, including independent validation studies are recommended.

## Competing interests

The author(s) declare that they have no competing interests.

## Authors' contributions

CA conceived and designed the study, oversaw all stages of data collection and analysis, and drafted the manuscript. GM coordinated all stages of the study, gave feedback on design, was responsible for data collection, supervised data entry, assisted with analysis and reviewed the manuscript. CE conducted focus groups, advised on qualitative methods, participated in consensus item selection processes and reviewed the manuscript. BC provided clinical advice on design and implementation of the study, assisted with recruitment, participated in consensus item selection processes and reviewed the manuscript. JC provided clinical advice on design and implementation, assisted with recruitment and reviewed the manuscript. SC assisted with analysis and reviewed the manuscript. JP, JLG, JG, PF and YS provided clinical advice on design and implementation, research advice on validation measures, assisted with recruitment and reviewed the manuscript. LS and KEB assisted with ethics approval processes, recruitment and data collection, and reviewed the manuscript. All authors read and approved the final manuscript.
